# Current Stroke Solutions Using Artificial Intelligence: A Review of the Literature

**DOI:** 10.3390/brainsci14121182

**Published:** 2024-11-26

**Authors:** Omar M. Al-Janabi, Amro El Refaei, Tasnim Elgazzar, Yamama M. Mahmood, Danah Bakir, Aryan Gajjar, Aysha Alateya, Saroj Kumar Jha, Sherief Ghozy, David F. Kallmes, Waleed Brinjikji

**Affiliations:** 1 Department of Neurology, Baptist Health, Lexington, KY 40503, USA; omar.al-janabi@bhsi.com; 2 Department of Neurology, Medical College of Wisconsin, Milwaukee, WI 53226, USA; 3 College of Medicine, Alfaisal University, Riyadh 11533, Saudi Arabia; 4 Central Pharmacy, University of Kentucky Healthcare, Lexington, KY 40536, USA; 5 Department of Neurology, School of Medicine, Southern Illinois University, Springfield, IL 62901, USA; 6 Department of Radiological Sciences and Neurological Surgery, David Geffen School of Medicine, University of California, Los Angeles, CA 92093, USA; 7 School of Medicine, Royal College of Surgeons in Ireland, Adliya P.O. Box 15503, Bahrain; 8 Tribhuvan University Teaching Hospital, Kathmandu 44600, Nepal; 9 Departments of Neurology and Neurologic Surgery, Mayo Clinic, Rochester, MN 55905, USA; 10 Department of Radiology, Mayo Clinic, Rochester, MN 55905, USA

**Keywords:** acute ischemic stroke, hemorrhagic stroke, large vessel occlusion, artificial intelligence, deep learning, automated detection

## Abstract

Introduction: In recent years, artificial intelligence (AI) has emerged as a transformative tool for enhancing stroke diagnosis, aiding treatment decision making, and improving overall patient care. Leading AI-driven platforms such as RapidAI, Brainomix^®^, and Viz.ai have been developed to assist healthcare professionals in the swift and accurate assessment of stroke patients. Methods: Following the PRISMA guidelines, a comprehensive systematic review was conducted using PubMed, Embase, Web of Science, and Scopus. Characteristic descriptive measures were gathered as appropriate from all included studies, including the sensitivity, specificity, accuracy, and comparison of the available tools. Results: A total of 31 studies were included, of which 29 studies focused on detecting acute ischemic stroke (AIS) or large vessel occlusions (LVOs), and 2 studies focused on hemorrhagic strokes. The four main tools used were Viz.ai, RapidAI, Brainomix^®^, and deep learning modules. Conclusions: AI tools in the treatment of stroke have demonstrated usefulness for diagnosing different stroke types, providing high levels of accuracy and helping to make quicker and more precise clinical judgments.

## 1. Introduction

Stroke is the second leading cause of death in the world, being responsible for more than 5.5 million deaths every year, and the leading cause of disability among patients [1,2]. The mortality rate of stroke remains a constant concern for patients and healthcare providers as it equates to roughly 10% during the first 30 days following an ischemic stroke, rising to 40% by the end of the first year [3]. Developing technology to provide accurate and reliable diagnoses to detect strokes and ultimately improve patient outcomes is necessary. Technologies such as CT scans and MRIs are essential, but interpretation by an experienced neuroradiologist may not be an option in hospitals in rural areas, which necessitates a more automated readily available AI tool to aid in this regard. In recent years, AI has played a significant role in transforming the care of stroke patients [4]. With the help of AI platforms such as RapidAI, Brainomix^®^, and Viz.ai, physicians can detect patients with stroke more efficiently and accurately. Additionally, it has facilitated better decision making and efficiency, leading to improved patient outcomes [5]. AI platforms and tools such as RapidAI, Brainomix^®^, and Viz.ai have significantly impacted the field. They will continue to play a transformative role in assisting medical professionals to improve decision making and the management of patients with stroke.

The three platforms, RapidAI, Brainomix^®^, and Viz.ai, bring unique technology and abilities to improve stroke detection. Firstly, RapidAI, developed by iSchemaView, has improved the field with advanced imaging software capable of instantly processing CT and MRI data. Moreover, their algorithms provide computerized maps of several parameters, including mean transit time, cerebral blood volume, and flow. These variables play a vital role in detecting and recognizing the penumbra (possibly recoverable tissue) and the ischemic core (irreversibly damaged tissue), allowing physicians to make decisions quickly during the acute stage of managing a stroke [6,7,8]. Secondly, Brainomix^®^ provides automated medical software to detect stroke. With the help of specific tools such as e-ASPECTS, the software can automatically evaluate CT scans without contrast to detect any initial ischemic changes [9,10]. Additionally, it improves evaluating thrombectomy eligibility by allowing healthcare providers to detect LVOs with CT angiography and providing CT perfusion analysis [10]. Lastly, Viz.ai utilizes deep learning algorithms to provide quick and improved detection of strokes while also providing LVO detection by utilizing CT angiography. Its quick and efficient detection platform, paired with user-friendly features, provides healthcare providers with easy communication and reduced time in manual detection [11].

AI is used in stroke solutions through platforms like RapidAI, Brainomix^®^, and Viz.ai and has provided healthcare providers with an efficient and improved method of detecting strokes and appropriate acute ischemic stroke management [12]. Additionally, these platforms have improved decision making over shorter periods and improved the efficiency and accuracy of detection, contributing to an overall improvement in patient outcomes [13]. As AI platforms continue to significantly improve stroke detection, they improve detection, accuracy, accessibility, and personalized treatment for patients. The further development of AI platforms will play a pivotal role in transforming the care of stroke patients by reducing mortality and improving quality of life.

## 2. Methods

### 2.1. Search Strategy

A literature review was conducted within the Nested Knowledge Autolit software version 1.46 (Nested Knowledge, Saint Paul, MN, USA), using PubMed, Embase, and Scopus. The search was conducted from the inception of the database until 30 June 2024. Based on each database, different combinations of possible keywords and/or Medical Subject Headings terms were used for that purpose.

**Figure 1:**
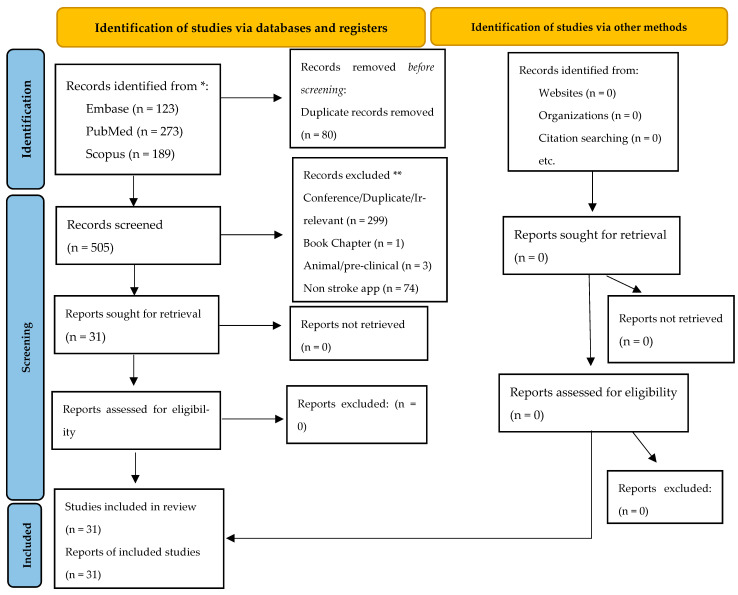
PRISMA flowchart detailing the literature review process. * Consider, if feasible to do so, reporting the number of records identified from each database or register searched (rather than the total number across all databases/registers). ** If automation tools were used, indicate how many records were excluded by a human and how many were excluded by automation tools. Source: Page MJ et al. [14].

Keywords and Medical Subject Headings terms included stroke, artificial intelligence, machine learning, deep learning, Viz.ai, RapidAI, and Brainomix^®^, besides others. The entire search strategy is provided in Supplement Table S1. An extensive manual search was performed through the references of the articles included to retrieve any missed papers. This study is conducted following the Preferred Reporting Items for Systematic Reviews and Meta-analyses (PRISMA) reporting guidelines (see Figure 1).

### 2.2. Screening Process

We included all original studies fulfilling our population, exposure, and outcome criteria, which totaled 31 studies (primarily retrospective cohort studies and one prospective study, one clinical trial, and one quality improvement study). We excluded studies if they met any of the following exclusion criteria:

Conference, duplicate, and irrelevant papers.
Reviews and meta-analyses.
Animal or pre-clinical studies.
Non-stroke AI applications.
Semi-automated applications.

### 2.3. Data Extraction

Following a pilot extraction, an extraction sheet was built, and the extraction was performed by 3 authors (TE., YM., and OA.). The extracted data included study characteristics and types of AI stroke applications together with sensitivity, specificity, and accuracy data for each AI stroke application as available. After performing the extraction, OA. and YM. conducted an extensive revision of the extracted data to avoid any mistakes or duplicate data.

## 3. Results

We included 31 studies, of which 29 discussed the use of stroke AI applications for detecting acute ischemic stroke (AIS), and two discussed the use of AI applications in hemorrhagic strokes. AI applications included mainly Viz.ai, Brainomix^®^, and RapidAI, among others. Refer to Table 1 for details.

### 3.1. Viz.ai

Multiple studies discussed the use of Viz.ai in stroke management. In a study by Hassan et al. [13], implementing Viz.ai to detect LVOs significantly reduced door-to-needle time from 132.5 min to 110 min, highlighting its impact on treatment timelines in LVO cases involving 43 patients. Martinez-Gutierrez et al. [21] reported a reduction in door-to-groin puncture time from 100 min to 88 min with AI-enabled LVO detection, emphasizing its efficiency in a cluster randomized clinical trial with 243 patients.

In addition, Figurelle et al. [23] found that Viz.ai improved door-to-groin time, showcasing its potential to enhance procedural efficiency in an initiative involving 82 patients. Karamchandani et al. [11] demonstrated high specificity (0.97) and moderate sensitivity (0.782) for Viz.ai LVOs, with an AUC of 0.88 and overall accuracy of 0.959 in detecting LVOs among 3851 patients.

Of note, Alwood et al. [25] compared Viz.ai with RapidAI, noting that Viz.ai provided larger core (25.9 cc) and penumbra (102.4 cc) estimates, underscoring its precision in 362 patients.

Finally, Bushnaq et al. [41] found that Viz.ai accurately predicted higher ischemic core volumes, comparable to RapidAI, in a retrospective multicenter study involving 129 patients.

Overall, Viz.ai has proven to be a valuable tool in stroke management, enhancing diagnostic accuracy, reducing treatment times, and improving clinical decision-making processes, thereby contributing to better patient outcomes.

### 3.2. RapidAI

RapidAI implementation in stroke studies has shown considerable advancements in diagnostic accuracy and clinical efficiency. In the study by Delora et al. [22], RapidAI demonstrated a sensitivity of 87% for detecting large vessel occlusions, comparable to Viz LVO, which had a specificity of 94%, effectively aiding in accurate occlusion identification in 360 patients.

In addition, Mallon et al. [24] compared Brainomix^®^ and RapidAI, finding that RapidAI accurately estimated ischemic core and penumbra volumes, with 22 mL for the core and 49 mL for the penumbra, across 90 patients. Alwood et al. [25] noted that RapidAI estimated core volumes at 18.2 cc and penumbra volumes at 84.6 cc, highlighting its precision in LVO cases involving 362 patients.

Of note, Chan et al. [33] reported high sensitivity for RAPID ASPECTS (87.5%) and RAPID CTA (92.3%), contributing to improved stroke assessment in 104 patients. Soun et al. [31] observed that implementing RAPID LVO significantly enhanced radiology workflow efficiency and accuracy in detecting acute ischemic stroke, with 96% sensitivity in a cohort of 760 patients. Schlossman et al. [37] found that RAPID LVO had higher sensitivity compared to CINA LVO, emphasizing its reliability in large vessel occlusion diagnosis in 263 patients.

Hoelter et al. [38] demonstrated that RapidAI, along with Syngo.via and Brainomix^®^, provided robust performance in AIS assessment, with a combined sensitivity of 0.777 and specificity of 0.734 across 131 patients. Similarly, Slater et al. [40] confirmed that experienced readers using RapidAI achieved higher sensitivity and specificity, reinforcing its diagnostic value in 500 patients. Bushnaq et al. [41] showed that RapidAI predicted ischemic core volumes with high accuracy, comparable to Viz.ai, in a multicenter study involving 129 patients. Finally, Pisani et al. [42] observed substantial agreement among RapidAI, Viz CTP, and e-CTP, underscoring its reliability in AIS management in 242 patients.

Overall, RapidAI has proven to be a critical tool in the diagnosis of stroke, providing high sensitivity and specificity, improving workflow efficiency, and ensuring accurate assessment of ischemic core and penumbra volumes.

### 3.3. Brainomix^®^

Several studies discussed the efficacy of Brainomix^®^ in enhancing diagnostic precision and improving patient outcomes. For instance, the research conducted by Weyland et al. [15] demonstrated Brainomix^®^‘s ability to detect hyperdense artery signs (HASs) in acute ischemic stroke (AIS) with a sensitivity of 0.77, 0.80, and 0.93 for software, reader 1, and reader 2, respectively, and specificity of 0.87, 0.97, and 0.71 for software, reader 1, and reader 2, respectively. This resulted in an AUC of 0.85 for software, 0.88 for reader 1, and 0.83 for reader 2, aiding in the automated detection and estimation of thrombus burden in 154 patients. In their research, Schmitt et al. [18] examined 160 patients and discovered that Brainomix^®^ effectively evaluated intracranial hemorrhage (ICH) with a sensitivity of 0.91 and specificity of 0.89, delivering precise diagnoses. They also found a sensitivity of 0.98 and a specificity of 0.89 for intraparenchymal hemorrhage (IPH). Mallon et al. [24] found that Brainomix^®^ and RapidAI combined showed improved accuracy in identifying ischemic stroke, 77% versus RapidAI’s 71%, in a study of 90 patients. In a prospective evaluation by Mallon et al. [27], Brainomix^®^ e-Stroke achieved a sensitivity of 58.6%, a specificity of 83.5% and accuracy of 77% for acute ischemic stroke, with strong correlations in the perfusion data for both core and penumbra regions, enhancing rapid and reliable diagnosis in 551 patients. Vacek et al. [28] highlighted the efficacy of Brainomix^®^ e-ASPECTS in delineating ICH, with 71% of cases rated as excellent or good in a cohort of 628 patients. A comparative study by Hoelter et al. [38] found that Brainomix^®^ had a sensitivity of 0.871 and specificity of 0.759, outperforming other tools like Syngo.via and RapidAI in assessing acute ischemic stroke in 131 patients.

Finally, in a retrospective study involving multiple centers led by Shahrouki et al. [43], the e-Stroke Suite (Brainomix^®^) was shown to accurately estimate ischemic core volumes on both non-contrast CT (NCCT) and CT perfusion (CTP), with average volumes of 20.4 mL and 19.9 mL, respectively, in 111 patients. In general, Brainomix^®^ has demonstrated its worth as a valuable tool for diagnosing different stroke types, providing high levels of accuracy, and helping to make quicker and more precise clinical judgments.

### 3.4. Deep Learning

The study by Chen et al. [33] demonstrated promising advancements in diagnostic accuracy by using deep learning models in stroke care. This study, conducted at multiple centers, concentrated on AIS and included 1476 patients, with 1391 in the development group and 85 in the validation group. When combined with conventional diagnostic methods, the deep learning model showed a sensitivity of 0.333 and a strong specificity of 0.915.

The model’s accuracy, assessed through the area under the curve (AUC), reached 0.876 internally and 0.729 externally, demonstrating consistent performance across various datasets. Even with lower sensitivity, the deep learning model is highly specific, indicating its effectiveness in accurately detecting non-stroke instances and decreasing false positives.

To date, research has found that the deep learning model greatly improves diagnostic capabilities for emergency doctors, helping them identify acute ischemic strokes accurately and efficiently. The enhanced diagnostic abilities highlight the potential of deep learning models to aid in clinical decision making and enhance patient outcomes in stroke treatment.

## 4. Discussion

While no amount of technology can truly replace a practicing physician in assessing patient symptoms and integrating the clinical context with appropriate management, artificial intelligence has proven beneficial in supplementing the delivery of quality stroke care to patients in the acute setting. The AI platforms summarized in this report, namely RapidAI, Brainomix^®^, and Viz.ai, provide unique technology that providers can use to assess stroke cases in a timely manner. There is not a great deal of data on the impact of these AI systems in the real-world management of acute stroke patients given that they are new and have mostly demonstrated theoretical applications. This article summarizes the current data regarding how AI systems can improve stroke management and deliver the best patient care in acute situations.

From the literature reviewed in this publication, it is clear that AI significantly improves the efficiency of care for patients in the high-acuity setting of ischemic stroke. In summary, the studies reviewed showed AI is able to significantly reduce door-to-needle time due to the improved detection of LVOs and more accurately estimate penumbra and infarct core volumes, enabling interventionalists to make a safer decision in relation to thrombectomy and more sensitively detect hemorrhages that may otherwise be missed on initial imaging.

While invaluable, traditional diagnostic imaging tools such as CT and MRI scans are subject to high reader variability amongst neurologists, interventionalists, and radiologists. Not only is the detection and characterization of the stroke necessary, but it must be realized in a timely manner, given the acuity of ischemic strokes. With AI aiding in recognizing early ischemic changes in imaging, clinicians can act faster. By creating a standardized technological system for detecting LVOs, determining ASPECT scores and other useful criteria for managing an acute stroke case, clinicians can be more reassured that they are delivering the best possible patient care [18,20].

The rapid advancements in AI have significantly impacted the assessment and management of acute ischemic stroke. Numerous artificial intelligence technologies, including Brainomix^®^, Viz.ai, and RapidAI, have shown an impressive ability in expediting the stroke diagnostic and treatment process [41]. Similar to the Viz.ai system, RapidAI has demonstrated remarkable performance, achieving 87% sensitivity in detecting large vessel occlusions (LVOs). Furthermore, RapidAI accurately estimated ischemic core and penumbra volumes, with mean estimates of 22 mL for the core and 49 mL for the penumbra. In the assessment of strokes, the RapidASPECTS and RapidCTA instruments also exhibited high sensitivity, at 87.5% and 92.3%, respectively. RapidLVO’s implementation improved radiology workflow accuracy and efficiency, resulting in a 96% sensitivity in identifying acute ischemic stroke.

The limitations of this review include the lack of cost of implementation and its effect on reducing cost for the patient and healthcare system in the setting of acute stroke management. Also, this study did not include studies that explored AI in stroke rehabilitation and long-term care. Finally, this study lacks longitudinal data to explore causality. Ultimately, more data are required regarding how AI would affect real-world workflow and whether the software constitutes more risk than benefit, as this information is limited. However, as seen in the available literature reviewed thus far, AI proves promising as a valuable tool in improving the management of acute ischemic stroke, ultimately optimizing patient outcomes. The widespread adoption of these AI-based technologies has led to faster decision making, reduced door-to-treatment times, and improved overall quality of care for patients suffering from acute ischemic stroke. With AI tools becoming widely available, future studies should focus on overcoming the challenges we are facing with the current tools together with strategies to maximize their wider implementation in stroke care beyond the acute stroke setting.

## Figures and Tables

**Table 1:** Main characteristics and outcome measures of the included studies.

Author(s) and Year	Study Design/Type	AI Tool	Sensitivity	Specificity	Accuracy	Door-to-Needle Time	Sample Size
Weyland et al., 2022 [15]	Retrospective Cohort Study	Brainomix^®^	0.77 (Software), 0.80 (Reader 1), 0.93 (Reader 2)	0.87 (Software), 0.97 (Reader 1), 0.71 (Reader 2)	N/A	N/A	154
Fasen et al., 2022 [16]	Retrospective Cohort Study	StrokeViewer	77.3% (AI), 78.7% (Manual)	88.5% (AI), 100% (Manual)	N/A	N/A	474
Gunda et al., 2022 [17]	Retrospective Cohort Study	e-Stroke Suite	N/A	N/A	N/A	42 min (2018), 44 min (2017)	797
Schmitt et al., 2022 [18]	Retrospective Cohort Study	Brainomix^®^	0.91 (ICH), 0.98 (IPH)	0.89 (ICH), 0.89 (IPH)	N/A	N/A	160
Seker et al., 2022 [19]	Retrospective Cohort Study	e-CTA	0.84	0.96	0.89	N/A	301
Hassan et al., 2020 [20]	Retrospective Cohort Study	Viz.ai LVO	N/A	N/A	N/A	110 min (post-AI), 132.5 min (pre-AI)	43
Martinez-Gutierrez et al., 2023 [21]	Cluster Randomized Clinical Trial	Viz.ai	N/A	N/A	N/A	100 min (pre-AI), 88 min (post-AI)	243
Delora et al., 2024 [22]	Retrospective Cohort Study	Viz LVO, Rapid LVO	0.87 (both)	0.96 (Viz), 0.85 (Rapid)	N/A	N/A	360
Mair et al., 2023 [10]	Retrospective Cohort Study	e-CTA	0.72	0.72	0.72	N/A	668
Figurelle et al., 2023 [23]	Quality Improvement Initiative	Viz.ai	N/A	N/A	N/A	N/A	82
Karamchandani et al., 2023 [11]	Retrospective Cohort Study	Viz.ai LVO	78.20%	97%	95.90%	N/A	3851
Mallon et al., 2022 [24]	Retrospective Cohort Study	Brainomix^®^, RapidAI	N/A	N/A	77% (Brainomix^®^), 71% (RapidAI)	N/A	90
Alwood et al., 2024 [25]	Multicenter Comparison	Viz.ai, RAPID.AI	N/A	N/A	N/A	N/A	362
Scavasine et al., 2024 [26]	Retrospective Cohort Study	e-CTA	N/A	N/A	N/A	N/A	97
Mallon et al., 2023 [27]	Prospective Evaluation Study	Brainomix e-Stroke	e-ASPECTS: 58.6%	e-ASPECTS: 83.5%	e-ASPECTS: 77.0%	N/A	551
Vacek et al., 2024 [28]	Retrospective Cohort Study	Brainomix e-ASPECTS	N/A	N/A	Excellent/Good: 71%, Moderate/Poor: 29%	N/A	628
Chan et al., 2022 [29]	Retrospective Cohort Study	RAPID ASPECTS, RAPID CTA	RAPID ASPECTS: 87.5%, RAPID CTA: 92.3%	RAPID ASPECTS: 30.9%, RAPID CTA: 85.3%	RAPID ASPECTS: 51.1% false positives	N/A	104
Mohapatra et al., 2023 [30]	Retrospective Cohort Study	VGG16 CNN	N/A	N/A	95.60%	N/A	517
Soun et al., 2023 [31]	Retrospective Cohort Study	RAPID LVO	0.96	0.85	N/A	N/A	760 (Pre-AI: 439, Post-AI: 321)
Lee et al., 2023 [32]	Clinical Validation Trial	Heuron ASPECTS	62.78% (ROI), 94.01% (>4 vs. ≤4), 95.42% (>6 vs. ≤6)	96.63% (ROI), 61.90% (>4 vs. ≤4), 76.56% (>6 vs. ≤6)	N/A	N/A	326
Chen et al., 2022 [33]	Multicenter Study	Deep Learning Model	0.333 (with AI)	0.915 (with AI)	N/A	N/A	1476 (1391 development, 85 validation)
Reidler et al., 2021 [34]	Retrospective Cohort Study	Automated Attenuation Measurements	0.87–0.91	0.97–0.99	N/A	N/A	145
Stib et al., 2020 [35]	Multicenter Retrospective Study	Convolutional Neuronal Network	1.00	0.77	N/A	N/A	540
Yahav-Dovrat et al., 2021 [36]	Retrospective Study	Viz LVO	0.81 (Overall), 0.82 (Stroke protocol)	N/A	0.94 (Overall), 0.89 (Stroke protocol)	N/A	1167
Schlossman et al., 2022 [37]	Retrospective Single-Center Study	RAPID LVO, CINA LVO	RAPID: 0.90, CINA: 0.76	RAPID: 0.86, CINA: 0.98	RAPID: 0.86, CINA: 0.96	N/A	263
Hoelter et al., 2020 [38]	Retrospective Study	Syngo.via, Brainomix^®^, RAPID	Syngo.via: 0.801, Brainomix^®^: 0.871, RAPID: 0.777	N/A	N/A	N/A	131
Sawicki et al., 2021 [39]	Retrospective Cohort Study	e-CTA	67% (Overall), 84% (Proximal), 36% (Distal)	95%	77%	N/A	108
Slater et al., 2024 [40]	Retrospective Study	RapidAI	LVO: 0.62, LVO/MVO: 0.39	LVO: 0.93, LVO/MVO: 0.92	LVO: 8.49, LVO/MVO: 5.0	N/A	500
Bushnaq et al., 2024 [41]	Retrospective Multicenter Study	RapidAI, Viz.ai	N/A	N/A	N/A	N/A	129
Pisani et al., 2023 [42]	Retrospective Multicenter Study	RAPID, Viz CTP, e-CTP	N/A	N/A	N/A	N/A	242
Shahrouki et al., 2023 [43]	Retrospective Multicenter Study	e-Stroke Suite (Brainomix^®^)	N/A	N/A	N/A	N/A	111

## Data Availability

Data are contained within the article or Supplementary Materials.

## Supplementary Materials

The following supporting information can be downloaded at: https://www.mdpi.com/article/10.3390/brainsci14121182/s1, Table S1: PRISMA 2020 Main Checklist. Reference [14] is cited in the supplementary materials.
